# Adipose Tissue T Cells in HIV/SIV Infection

**DOI:** 10.3389/fimmu.2018.02730

**Published:** 2018-12-03

**Authors:** Celestine N. Wanjalla, Wyatt J. McDonnell, John R. Koethe

**Affiliations:** ^1^Division of Infectious Diseases, Department of Medicine, Vanderbilt University Medical Center, Nashville, TN, United States; ^2^Center for Translational Immunology and Infectious Disease, Vanderbilt University Medical Center, Nashville, TN, United States; ^3^Department of Pathology, Microbiology, and Immunology, Vanderbilt University, Nashville, TN, United States

**Keywords:** HIV—human immunodeficiency virus, SIV–simian immunodeficiency virus, obesity, adipose tissue, immunology, inflammation, stromal vascular fraction

## Abstract

Adipose tissue comprises one of the largest organs in the body and performs diverse functions including energy storage and release, regulation of appetite and other neuroendocrine signaling, and modulation of immuity, among others. Adipocytes reside in a complex compartment where antigen, antigen presenting cells, innate immune cells, and adaptive immune cells interact locally and exert systemic effects on inflammation, circulating immune cell profiles, and metabolic homeostasis. T lymphocytes are a major component of the adipose tissue milieu which are altered in disease states such as obesity and human immunodeficiency virus (HIV) infection. While obesity, HIV infection, and simian immunodeficiency virus (SIV; a non-human primate virus similar to HIV) infection are accompanied by enrichment of CD8^+^ T cells in the adipose tissue, major phenotypic differences in CD4^+^ T cells and other immune cell populations distinguish HIV/SIV infection from obesity. Furthermore, DNA and RNA species of HIV and SIV can be detected in the stromal vascular fraction of visceral and subcutaneous adipose tissue, and replication-competent HIV resides in local CD4^+^ T cells. Here, we review studies of adipose tissue CD4^+^ and CD8^+^ T cell populations in HIV and SIV, and contrast the findings with those reported in obesity.

## Introduction

The adipose tissue stromal vascular fraction (SVF) contains a diverse range of innate and adaptive immune cells, which interact in a complex paracrine signaling environment that affects adipocyte energy storage and other functions. T lymphocytes, or T cells, are a major component of the adipose tissue environment recruited from the circulatory and lymphatic systems, and these T cell populations undergo marked changes with progressive adiposity in overweight and obese persons. More recently, a handful of studies have identified substantial changes in adipose tissue T cell density and cellular characteristics in human immunodeficiency virus (HIV) and simian immunodeficiency virus (SIV; a non-human primate virus similar to HIV) infection, including shifts in total CD4^+^ and CD8^+^ T cells and subsets, cytokine production, antigen specificity, interactions with adipocytes and other SVF cells, and capacity for latent infection with HIV or SIV proviruses (1–6). These studies, and their principal findings, are described in Table 1.

**Table 1:** Summary of studies on adipose tissue T cells and the viral reservoir in HIV and SIV.

**References**	**HIV/SIV**	**Subjects**	**Principal T cell findings**	**Principal viral reservoir findings**
Couturier et al. (1)	HIV	4 ART-treated HIV+ humans (3 alive, 1 cadaver) 1 ART-naïve HIV+ cadaver	•Higher CD8:CD4 ratio in AT compared to blood •AT CD4^+^ and CD8^+^ T cells predominantly CD69^+^ memory cells	•*Gag* and *Env* proviral DNA detected by nested PCR •Co-culture of latently HIV-infected CD4^+^ T cells with adipocytes and IL-2, IL-7, or IL-15 increased T cell activation and HIV production
Damouche et al. (2)	HIV and SIV	13 ART-treated, VL suppressed HIV+ humans 23 SIV+ cynomolgus macaques 21 SIV-negative cynomolgus macaques	HIV+: •none- study primarily assessed viral reservoir •[SIV^+^:] •Higher adipose tissue CD8:CD4 ratio in SIV+ vs. SIV-negative •Higher CD4^+^ and CD8^+^ T cell HLA-DR (activation) expression in SIV+ vs. SIV-negative •CD4^+^ and CD8^+^ predominantly CD95^+^ memory cells in AT •CD4^+^ predominantly CD69^+^ in AT (although co-expression of CD69 on memory T cells was not assessed) •Similar memory T cell subset distribution in SIV+ and SIV-negative •Higher inflammatory macrophages in SIV+ vs. SIV-negative	HIV: •HIV DNA detected in total SVF cells and sorted adipose tissue CD4^+^ T cells •Level of HIV DNA in CD4^+^ T cells similar to blood •HIV RNA detected by *in situ* tissue hybridization and after *in vitro* reactivation of CD4^+^ T cells •[SIV:] •SIV DNA present in SVF and in sorted CD4^+^ T cells and macrophages •SIV RNA detected by *in situ* tissue hybridization and in CD4^+^ T cells and macrophages
Couturier et al. (3)	SIV and SHIV	8 SHIV-SF162p3-infected rhesus macaques (acute) 8 SIVmac251-infected macaques (chronic) 7 non-infected macaques	•Higher adipose tissue CD8:CD4 ratio in SHIV+ vs. SHIV-negative •CD4^+^ and CD8^+^ predominantly CD95^+^ CD69^+^ memory cells •Similar levels of NKT cells in SHIV+ compared to SHIV-negative	•Infectious SIV inducible from SVF CD4 T cells;
Damouche et al. (4)	HIV	11 ART-treated HIV+ humans 19 HIV-negative humans	•Higher adipose tissue CD8:CD4 ratio in SAT, but not VAT, from HIV+ vs. HIV-negative •Higher proportion of Treg cells in SAT from HIV+ vs. HIV-negative •No difference in CD4^+^ Th1, Th2 and Th17 cell proportions in HIV+ vs. HIV-negative •No difference in CD4^+^ expression of Ki-67 or HLA-DR in HIV+ vs. HIV-negative •Higher CD4^+^ and CD8^+^ expression of PD-1 in AT vs. blood from HIV+ subjects •AT CD4^+^ and CD8^+^ T cells predominantly CD69^+^ cells	•None—study primarily assessed CD4^+^ T cell subsets
Hsu et al. (5)	SHIV	6 SHIV+ rhesus macaques	•Increase in adipose tissue CD4^+^ cells after SHIV infection (CD8^+^ not reported)	•Lower SHIV RNA in SAT compared to rectum and lymph node •SHIV RNA levels similar in SAT and VAT
Koethe et al. (6)	HIV	10 ART-treated, VL suppressed HIV+ humans	•Higher CD8:CD4 ratio in adipose tissue vs. blood •Higher CD8^+^ expression of HLA-DR and CD57 in adipose tissue •Higher CD8^+^ TCRβ clonality in adipose tissue; distinct TCRβ V and J gene usage •AT CD4^+^ and CD8^+^ T cells exhibited limited CD69 expression	•Level of HIV DNA in adipose tissue CD4^+^ T cells similar to blood
Couturier et al. (7)	HIV	8 HIV-infected AT specimens	•HIV-infected CD4^+^ T cells cultured with adipocytes and ART had suppressed viral replication and increased survival •After 7 days CD4^+^ p24^+^ T cells cultured with adipocytes had higher CD69 expression (~8–12%) compared to <2% CD69^+^ p24^+^ T cells if cultured in media alone	•CD4^+^ T cells from AT-SVF of 2/3 individuals produced infectious virus in an outgrowth assay •NNRTIs were less detectable in AT cells likely because they are hydrophilic. Dolutegravir was found in higher intracellular concentrations in AT

*ART, antiretroviral therapy; AT, adipose tissue; HIV, human immunodeficiency virus; SAT, subcutaneous adipose tissue; SHIV, simian-human immunodeficiency virus; SIV, simian immunodeficiency virus; SVF, stromal vascular fraction; TCR, T cell receptor; VAT, visceral adipose tissue*.

In general, mammals have predominantly white adipose tissue in their viscera and subcutaneous fat depots. While the accumulation of visceral adipose tissue (VAT) generally has greater detrimental effects on metabolic health, T cell subsets in subcutaneous adipose tissue (SAT) and VAT appear similar from an immune perspective. Paired SAT and VAT samples from SIV-infected macaques showed no statistically significant differences in the percentage of CD4^+^ T cells, activated HLA-DR^+^ T cells, or CD69^+^ memory T cells (2). Furthermore, paired SAT and VAT samples from 47 surgical patients showed close correlation between the percentage of CD8^+^ cells (*r* = 0.90, *p* < 0.01), CD4^+^ cells (*r* = 0.90, *p* < 0.01), T_H_17 cells (*r* = 0.75, *p* = 0.01), and T_H_1 cells (*r* = 0.67, *p* < 0.04) (8).

In contrast to SAT and VAT, brown fat is mainly supraclavicular, paravertebral and suprarenal (9–11). While white adipose tissue primarily functions as an energy store, brown adipocytes have more mitochondria and are involved in energy expenditure and thermogenesis. The latter may replace white adipocytes after thermogenic stimulation (12). Beige adipocytes are a third group that demonstrate a functional resemblance to brown adipocytes. They contain high levels of mitochondria and may be derived from white adipocytes (13, 14). Obese persons have less brown adipose tissue compared to their lean counterparts, and brown adipose tissue generally contains fewer immune cells compared to white adipose tissue. These distinctions of function and location are important to contextualize studies on the role of the immune system in adipose tissue. At present, the majority of studies of adipose tissue T cells in HIV and SIV are representative of white adipose tissue physiology from the SAT and VAT compartments.

An enrichment of adipose tissue CD8^+^ T cells and an increase in the CD8:CD4 ratio accompanies HIV and SIV infection, which is a phenomenon also observed in obesity. However, adipose tissue changes in HIV should not be considered “equivalent” to obesity, as marked differences in CD4^+^ T cell and macrophage profiles are present in the two conditions. It is thought that several mechanisms drive both CD8^+^ T cell enrichment and the shifts in T cell distribution in obesity. Several chemokines are detected in obese adipose tissue, including CXCL10, CXCL8, CCL5, and CCL2 (15–17). At present, there is a paucity of data on chemokine receptor expression on adipose tissue T cells, though these T cells can infiltrate inflamed adipose tissue via chemotactic recruitment by CCL5/RANTES and interaction with CXCR4 and CCR5 (18). Notably, CCL20 expression by human adipocytes is higher in obese individuals (19). Finally, when discussing adipose tissue immunology in HIV infection, it is paramount to consider the impact of HIV DNA and RNA in the local environment on T cell subset profiles and cellular function.

## Adipose tissue T cell changes in HIV/SIV

### Increase in the adipose tissue CD8:CD4 T cell ratio in HIV and SIV

**Figure 1:**
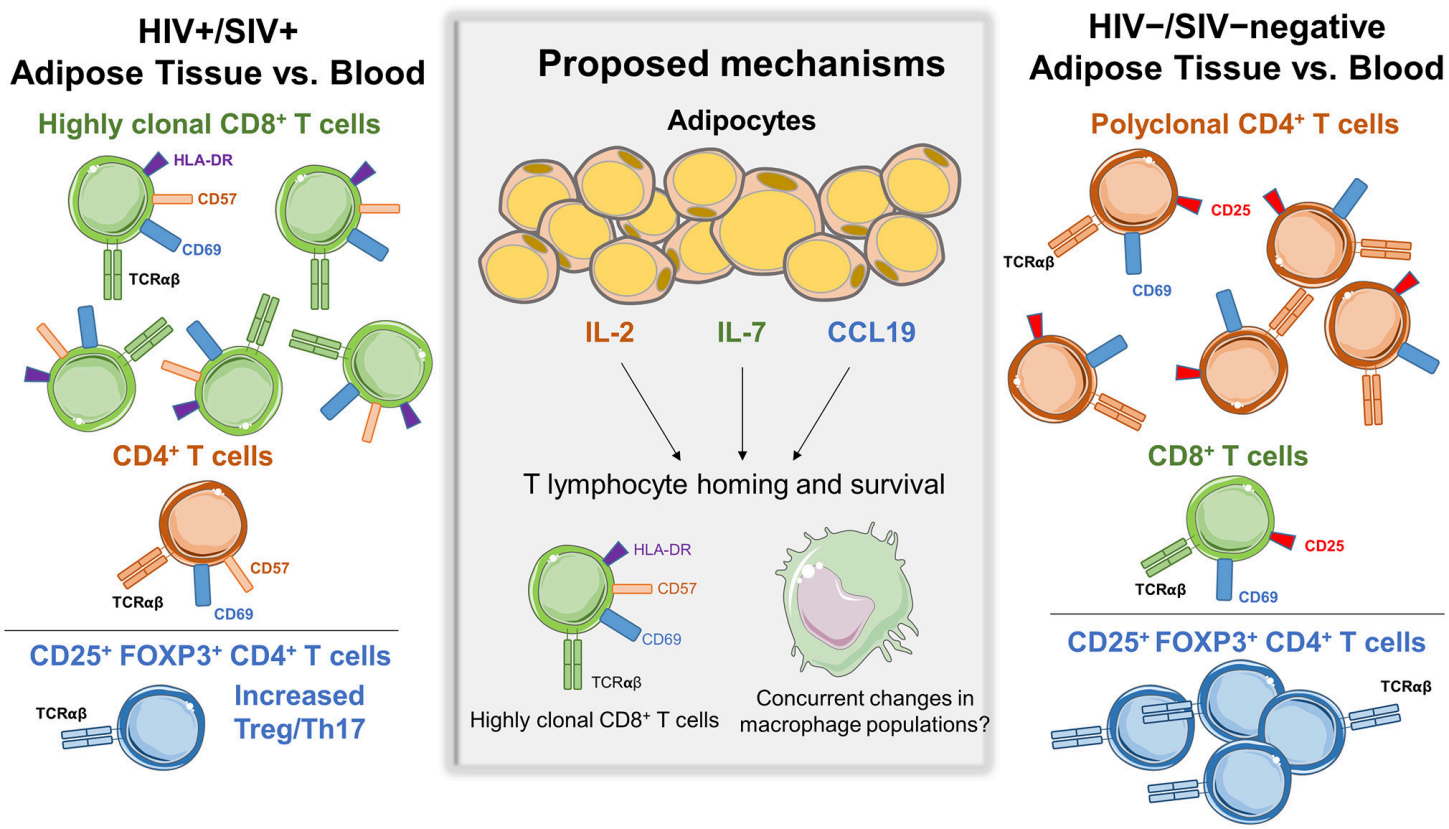
Differences in adipose tissue and blood CD4^+^ and CD8^+^ subsets in HIV/SIV+ vs. HIV/SIV-negative subjects. Adipose tissue in HIV/SIV+ subjects is enriched in CD8^+^ T cells compared to blood, while HIV/SIV-negative adipose tissue is enriched in CD4^+^ T cells. A proposed mechanism is the preferential recruitment of cells from circulatory/lymphatic systems via differences in cytokine expression by adipocytes and adipose tissue stromal vascular fraction cells in HIV/SIV+ including IL-2, IL-6, IL-7, IL-8, CCL19, IL-12p40, and MIP-1α, eotaxin and interferon gamma. While a study in HIV+ subjects found increased CD8^+^ T cell clonality in adipose tissue, there is currently not compelling evidence of *in situ* CD4^+^ or CD8^+^ T cell proliferation within adipose tissue.

One of the first studies of T cells in the SAT and VAT of persons living with HIV (PLWH), by Couturier et al., identified major differences in CD4^+^ and CD8^+^ T cell populations compared to HIV-negative controls (1). Similar findings were subsequently reported in other HIV and SIV studies (2, 4, 6). Adipose tissue was collected from 3 living and 2 deceased PLWH, and 4 healthy controls. Cells within the SVF were isolated by collagenase digestion, separated by Ficoll gradient, and analyzed by flow cytometry. The adipose tissue SVF CD3^+^ T cells were predominantly memory CD4^+^ CD45RO^+^ T cells (61%) in the HIV-negative controls, with fewer memory CD8^+^ T cells (15%). Furthermore, the proportion of memory CD4^+^ T cells in adipose tissue of healthy controls was ~50% higher compared to blood (1). In contrast, this distribution was reversed in PLWH, with more adipose tissue memory CD8^+^ T cells (46%) than memory CD4^+^ T cells (35%), which represented an ~50% enrichment in memory CD8^+^ T cells over the blood and could not be attributed to depletion of circulating CD4^+^ T cells. Figure 1 summarizes the major differences in CD8^+^ and CD4^+^ T cell profiles in adipose tissue and blood from HIV/SIV-infected subjects vs. HIV/SIV-negative controls (described in further detail below).

A study of 10 PLWH on the same antiretroviral therapy (ART) regimen (efavirenz, tenofovir, emtricitabine) with long-term virologic suppression by Koethe et al. found a higher proportion of total CD8^+^ T cells in SAT compared to blood (6). This study was limited by a lack of a HIV-negative comparison group. SAT samples were obtained by liposuction biopsies and processed by collagenase digestion and Ficoll separation similar to Couturier et al. (1). SAT was enriched in CD8^+^ T cells (61%) over CD4^+^ (33%), as compared to 52 and 43% in the blood, respectively. Memory T cell proportions, defined as CD45RO^+^ CD8^+^ and CD4^+^ T cells, were not significantly different between SAT and blood.

As observed in PLWH, cynomolgus macaques with SIV infection have a higher percentage of CD8^+^ T cells, and lower CD4^+^ T cells, in both the SAT and VAT compared to non-infected animals (2, 3). In a study by Damouche et al. the proportion of CD8^+^ T cells was significantly higher in the SAT and VAT of SIV-infected macaques (2). Correspondingly, CD4^+^ T cell percentages were significantly lower (18% in SAT and 20% in VAT) compared to non-infected animals (40% in both SAT and VAT). Notably, this study demonstrated that the change in the CD8:CD4 ratio was not driven by a reduction in the total number of CD4^+^ T cells in infected animals. Rather, SIV-infected animals had significantly higher density of CD8^+^ T cells in VAT and a somewhat higher density in SAT (2). For both non-infected healthy and SIV-infected monkeys, nearly all of the adipose tissue CD4^+^ and CD8^+^ T cells were memory T cells (>94% CD95^+^), with a large fraction of activated cells marked by expression of CD69^+^ (62–84%) and CD25^+^ (3–13%). This seemingly CD8-mediated inversion of the typical CD4^+^ and CD8^+^ T cell ratio was also noted in PLWH in a separate study by Damouche et al. (4).

In a second study of macaques, a higher adipose tissue CD8:CD4 ratio was observed in animals infected with SIV 4-weeks prior (ratio 1.51) compared to non-infected animals (ratio 1.38) (3). Interestingly, the CD8:CD4 ratio was similarly elevated (1.59) in non-infected animals with chronic enterocolitis, suggesting chronic illness or inflammation may affect adipose CD8^+^ and CD4^+^ T cell trafficking independently of viral infection. Gene expression profiling on the adipocyte enriched floating fraction from SIV-infected animals showed higher levels of CCL19, interleukin (IL)-2, and IL-7, which may contribute to T cell homing and survival (3). Additional studies are needed to replicate these recent reports and elucidate a mechanism for enrichment of CD8^+^ T cells in adipose tissue of PLWH and SIV-infected macaques.

### CD57, CD69, HLA-DR and PD-1 expression on adipose tissue T cells

Recent studies have assessed CD57, CD69, HLA-DR, and PD-1 expression on T cells in the blood and adipose tissue of PLWH and SIV-infected macaques, and as compared to non-infected controls. Koethe et al. examined expression of CD57 on adipose tissue T cells from PLWH on long-term ART, as CD4^+^ and CD8^+^ T cell CD57 expression in blood is higher in PLWH compared to HIV-negative persons (20–22). CD57 is a terminally sulfated glycan carbohydrate epitope found on T cells and natural killer (NK) cells. CD57 is a marker of late differentiation, though there is limited consensus as to whether CD57 expression implies reduced replicative capacity, an inability to proliferate in response to antigen stimulation, or increased susceptibility to activation-induced apoptosis (20, 23, 24). CD8^+^ T cells expressing CD57 produce more interferon-γ and tumor necrosis factor-α (TNF-α) after T cell receptor (TCR) stimulation compared to CD8^+^CD57^−^ T cells, and demonstrate a distinct gene expression profile characterized by greater cytotoxic effector potential (e.g., production of perforin, granzymes, and granulysin) (25, 26). A higher percentage of CD57^+^ T cells is also implicated in several inflammatory diseases (27–29).The proportion of CD8^+^ T cells expressing CD57 was significantly higher in SAT compared to blood (37 vs. 23%, respectively) from PLWH, and activated CD8^+^ T cells expressing HLA-DR were over six-fold higher in SAT compared to blood (5.5 vs. 0.9%, respectively).

A notable difference between the Couturier and Koethe studies was the reported expression of CD69 on adipose tissue CD8^+^ and CD4^+^ T cells. CD69 is an inducible, early-activation indicator which serves as a putative tissue-resident marker on memory T cells in human mucosal and lymphoid tissues (30–32), but is largely absent on circulating blood memory T cells (31). In a study by Couturier et al., surface expression of the CD69 activation marker on adipose tissue memory CD4^+^ and CD8^+^ T cells was 67 and 60%, respectively, in the PLWH and 72 and 61%, respectively, in the HIV-negative, compared to <5% in the blood. In contrast, Koethe et al. found overall expression of CD69 was low (<5%) on both adipose tissue CD8^+^ and CD4^+^ T cells (though still higher than in blood), but among adipose tissue memory CD4^+^ T cells CD69 expression was 20-fold higher compared to blood (5.9 vs. 0.3%). The reason for this disparity is unclear and could include differences in CD69 antibody binding, time-related increases in CD69 expression in adipose tissue samples collected after death or by surgical resection as opposed to rapid T cell extraction from liposuction aspirates, an artifact introduced by collagen digestion or other aspects of processing, or greater residual contamination of liposuction aspirates by peripheral blood. Additional studies are needed to assess CD69 expression with different experimental methods, though a study comparing activation markers on adipose tissue T cells in lean, overweight and obese individuals using aspiration via a 14 G needle found ~5–10% CD69 expression on CD4^+^ T cells and ~25% expression on CD8^+^ T cells (33).

In a study by Damouche et al., expression of the HLA-DR activation marker on CD8^+^ and CD4^+^ T cells was significantly higher in SAT, VAT, and blood from SIV-infected macaques compared to non-infected animals (2). However, the authors did not observe a significant difference in the proportion of Ki-67-expressing SAT and VAT CD4^+^ or CD8^+^ T cells in SIV-infected animals compared to controls. Cycling and recently divided T cells express Ki-67, while resting cells do not. This suggests that differences in the activation profile, and the higher density of CD8^+^ T cells, in adipose tissue may not result from recent *in situ* proliferation. Another significant contribution of this study was the visualization of T cells in adipose tissue. Histology showed that CD4^+^ T cells were primarily located among adipocytes far from the capillaries, while CD8^+^ T cells clustered in the pericapillary area (2). The positioning of CD4^+^ T cells deeper in adipose tissue could serve to protect latently-infected CD4^+^ cells against cytotoxic killing by CD8^+^ T cells.

PD-1 (CD279) expression on CD4^+^ T cells is a negative co-stimulator that can inhibit TCR signaling (34, 35) and is important for mediating tissue tolerance of potentially autoreactive cells (36). In the context of HIV, cells expressing PD-1 could hinder clearance of chronic viruses by allowing tolerance due to their exhausted phenotype (37). Murine CD4^+^ memory T cells expressing CD44 and PD-1 were increased in VAT of high fat diet fed (HFD) mice (55%) compared to mice fed a normal diet (33%), and these cells expressed less IL-2 and IFN-γ compared to PD-1^−^ CD44^hi^ CD4^+^ T cells (38). Notably, the PD-1^+^ CD44^hi^ CD4^+^ T cells activated via the TCR also expressed large amounts of osteopontin (OPN), a negatively charged, N-glycosylated secreted phosphoprotein produced by numerous cell types, and higher levels of OPN were present in the serum of HFD mice. A fraction of the PD-1^+^ CD44^hi^ CD4^+^ T cells also expressed CD153 (CD30L), a type II membrane glycoprotein that is present primarily on activated CD4^+^ T cells, and the CD153^+^ PD-1^+^ CD44^hi^ CD4^+^ T cells were found to express genes linked to senescence (38). Adoptive transfer of these CD153^+^ PD-1^+^ CD44^hi^ CD4^+^ T cells into lean mice on a normal diet induced inflammation in VAT, as well as insulin resistance. This study suggests a role for PD-1 expressing T cells in metabolic disease.

Damouche et al. found high expression of PD-1 on CD4^+^ T cells in SAT (45%) and VAT (62%) compared to in peripheral blood (3%) (4). However, there were no significant differences in PD-1 expression between PLWH and HIV-negative individuals. The authors further demonstrated that PD-1 expression was higher in CD69^+^ CD4^+^ T cells compared to CD69^−^ CD4^+^ T cells within SAT and VAT of HIV-negative individuals (a similar analysis was not done in PLWH due to limited sample number). Additionally, this study found a higher proportion of TIGIT/PD-1^+^ CD4^+^ T cells in SAT compared to VAT, which the authors postulated may be important for HIV-1 persistence. This finding suggests differences between the two fat depots, and evaluation of TIGIT/PD-1^+^ cells in the context of the viral reservoir in PLWH is an area for future research.

### Increased CD8^+^ T cell receptor clonality in adipose tissue from PLWH

The higher CD8:CD4 ratio in adipose tissue from SIV-infected macaques was attributed to an increase in CD8^+^ T cells as opposed to depletion of CD4^+^ T cells in one study (2), though additional data are needed in humans. As CD8^+^ T cell density increases, key questions include: (1) whether recruitment of primarily activated and memory CD8^+^ T cells from the circulation is stochastic; (2) whether CD8^+^ T cells with select antigen specificities and corresponding TCR characteristics are preferentially migrating to adipose tissue; (3) whether CD8^+^ T cells are clonally expanding *in situ* in response to TCR stimulation; and (4) whether T cells that infiltrate adipose tissue re-enter circulation. The Koethe et al. study of PLWH with long-term virologic suppression found increased CD8^+^ TCR clonality in adipose tissue compared to blood using bulk TCRβ CDR3 deep sequencing, where bias-controlled V and J gene primers are used to amplify rearranged V(D)J segments (6, 39, 40). In all five subjects with adequate DNA for adipose tissue CD8^+^ TCR sequencing, the 10 most prevalent TCRβ clones comprised a significantly larger percentage of total clones in SAT (25%) compared to paired blood (16%), and the Shannon's Entropy index, a measure of overall repertoire diversity, was lower in adipose tissue compared to blood (4.39 vs. 4.46, respectively). Notably, the same TCRβ sequences, also referred to as public clonotypes (i.e., specific rearrangements observed in multiple individuals), were not observed to occur at >0.9% frequency in the adipose tissue among any two study subjects. Gene usage and V-J gene pairing differed between blood and adipose tissue, but these differences were not statistically significant, potentially due to the low number of subjects.

The finding of increased adipose tissue CD8^+^ T cell clonality in PLWH has correlates in the obesity literature (41–43). A notable finding from *in vitro* studies is that obese fat can independently activate CD8^+^ T cells and induce proliferation, while lean fat has little effect (41). In mice, Yang et al. observed shifts in the clonality of both the adipose tissue CD4^+^ and CD8^+^ TCR repertoires in lean vs. obese animals using PCR-based spectratyping, which was based on shifts in gene family expression from a normally Gaussian frequency distribution of CDR3 length (43). Winer et al. examined TCR Vα diversity and observed clonal expansion in SAT of CD4^+^ T cells of mice on a HFD (42). A major limitation of these studies from the obesity literature was the use of spectratyping, which can only provide indirect evidence of clonal expansion, cannot detect individual TCR sequences, and cannot distinguish between T cell clones sharing the same V gene region but distinct junctional regions. The introduction of TCR sequencing makes it possible to examine immune receptor repertoires with fine specificity, and to examine the genetic and biochemical properties of both individual TCRs and the broader repertoire.

McDonnell et al. recently evaluated liver and adipose tissue CD4^+^ and CD8^+^ TCR repertoires in mice fed a HFD or low fat diet. They performed high-throughput TCR sequencing, which allowed distinction of T cell clones and quantification of clonal expansion (44). The authors found that mice on a HFD had reduced CD8^+^ TCR diversity similar to previous studies (6, 39, 40). Moreover, they identified public TCR clonotypes and a predominance of positively-charged CDR3 regions with less polar amino acids. Notably, adipose tissue from mice on a HFD expressed elevated isolevuglandins (isoLG; a group of negatively charged reactive gamma-ketoaldehydes generated by free radical oxidation) in M2-polarized macrophages. Co-culture of these isoLG-bearing M2 macrophages with CD8^+^ T cells isolated from the spleens of obese mice on a HFD led to an increase in T cell proliferation and activation *in vitro*, and these T cells showed increased expression of CD69, a marker suggestive of TCR-linked activation. This was the first study to indicate macrophages presenting isoLG can promote CD8^+^ T cell activation, and may reflect a contribution of modified proteins to the propagation of inflammation within the adipose tissue of obese mice and humans.

The finding of a more clonal CD8^+^ T cell population in the adipose tissue of PLWH, and elevated CD4^+^ and CD8^+^ clonality in obesity, raises the question of where and how antigen might be presented to drive TCR clonal expansion. Several published reports indicate that antigen presentation occurs directly in adipose tissue, with a focus on CD4^+^ T cells. Several distinct MHC-II-expressing antigen presenting cells (APCs) in adipose tissue can present antigens to CD4^+^ T cells; these include B cells (45), dendritic cells (46), macrophages (47), and even adipocytes (48). Mice with global deficiency of MHC-II demonstrate protection from adipose tissue inflammation and systemic insulin resistance when placed on a HFD (47, 48); the accumulation of CD4^+^ T cells and reduction in CD11c^+^ macrophages may be responsible for the reduced adipose tissue inflammation in these animals (47). Cells in the adipose tissue also secrete adipokines such as leptin, adiponectin, and retinol binding protein 4 that can directly activate these APCs (49–51).

### Adipose tissue CD4^+^ T cell subsets in HIV and SIV

While HIV and SIV infection are characterized by an increased density of adipose tissue CD8^+^ T cells, the limited studies on CD4^+^ T cell subsets show less clear differences between PLWH and HIV-negative persons. A study by Damouche et al. focused on the CD4^+^ T cell compartment in adipose tissue from ART-treated PLWH and HIV-negative controls (4). As observed in the earlier study by Couturier et al. (1), HIV-negative subjects had a predominance of CD4^+^ T cells over CD8^+^ T cells in SAT (55 vs. 25%, respectively), but this ratio was inverted, to a degree, in the SAT of those with HIV (40 CD4^+^ vs. 50% CD8^+^ T cells). Interestingly, the difference in CD8:CD4 ratio according to HIV status was not observed in VAT. CD4^+^ and CD8^+^ T cell subsets were equally represented (43 and 41%, respectively) in VAT from HIV-negative subjects, and this relative proportion did not differ in the PLWH. This finding differs from a study of SIV-infected macaques, which found a significantly higher CD8:CD4 ratio in VAT from SIV-infected animals compared to controls (2).

Among CD4^+^ T cell subsets, Damouche et al. observed a significantly *higher* proportion of SAT regulatory T cells (Tregs; defined as CD25^+^ FOXP3^+^ CD4^+^ T cells) in the PLWH compared to HIV-negative subjects, but no major differences in T_H_1 and T_H_17 pro-inflammatory subsets (4). Treg cells, a subset that can be difficult to evaluate due to the technical aspects of staining for markers such as Foxp3, accounted for 7.2% of SAT CD4^+^ T cells in the PLWH, compared to 1.7% in the HIV-negative. A similar but non-significant difference was observed in VAT Treg cells. Obesity is commonly associated with *reduced* T regs in adipose tissue of obese humans and mice, and loss of these cells is postulated to facilitate the influx of pro-inflammatory T cells and macrophages (52). Furthermore, the authors did not observe a significant difference in T_H_17 cells, identified by expression of ROR-γ transcription factors, according to HIV status. The proportion of T_H_1 CD4^+^ T cells (cells with intermediate or high levels of T-bet expression) also did not differ according to HIV status, while T_H_2 CD4^+^ T cells (expressing GATA-3) were barely detected in the adipose tissue from both PLWH and HIV-negative subjects. Taken together, a unifying finding of these experiments is a higher Treg/T_H_17 ratio in the SAT of PLWH, potentially reflecting a compensatory, anti-inflammatory response (4).

In the same study, no significant differences were observed in the proportion of adipose tissue CD4^+^ or CD8^+^ T cells expressing either Ki-67 or HLA-DR between PLWH vs. HIV-negative controls (4). The median percentage of Ki-67^+^ cells was <2% in both SAT and VAT from PLWH and controls, potentially indicating limited T cell proliferation within the tissue. The expression of HLA-DR on CD4^+^ cells in SAT (24%) and VAT (21%) was higher compared to blood in the HIV-negative, but again similar values were observed in the PLWH. Finally, the authors observed far higher PD-1 expression on adipose tissue CD4^+^ T cells (45% expression in SAT and 62% in VAT) compared to peripheral blood (3%), but again there were no significant differences according to HIV status (4). Notably, PD-1 expression was primarily present on CD69^+^ CD4^+^ T cells, suggesting those cells thought to represent a “tissue resident” phenotype are also more likely to demonstrate the features of quiescent cells. The authors postulated that the high degree of exhaustion marker expression is indicative of limited T cell proliferation and activation, as evidenced by the low Ki-67 expression, thus reducing downstream macrophage activation and tissue inflammation. However, while the enrichment of PD-1^+^ CD4^+^ T cells could have a protective role in limiting inflammation, high PD-1 expression on adipose tissue T cells may be of particular importance in the context of HIV as these cells are thought to constitute a large part of the latent HIV reservoir (53–55). Studies to date have not yet measured Bcl-2, which will be important to clarify how susceptible these populations are to apoptosis.

## Comparison of adipose tissue cell changes in HIV/SIV and obesity

### T cells in HIV/SIV and obesity

The finding that adipose tissue from PLWH and SIV-infected macaques is enriched in CD8^+^ T cells was particularly intriguing as similar changes are a hallmark of obesity. Studies of obese humans and obese animal models show a striking increase in adipose tissue CD8^+^ T cells and CD4^+^ T_H_1 cells, a decrease in Treg cells, and an increase in M1-phenotype (TNF-α, IL-6, IL-12, IL-23-producing) pro-inflammatory macrophages compared to non-obese controls (41, 42, 45, 52). A landmark 2009 paper by Nishimura et al. showed the recruitment of M1-phenotype macrophages into adipose tissue in mice was dependent on the prior infiltration of CD8^+^ T cells (41).

CD4^+^ Treg cells protect against tissue inflammation and insulin resistance, and are less frequent in obese adipose tissue compared to lean. Adipose tissue Tregs in obese mice have a unique TCR profile compared to Tregs from the spleen and lymph nodes, as evidenced by single cell sequencing from each of these compartments (52). An important caveat of this study was that the “limited” Ltd transgenic mice used can only generate a highly restricted Vα repertoire that pairs with a single TCRβ chain (56). Experimental models demonstrate that the adoptive transfer of CD4^+^ T cells into lymphocyte-free Rag1^−/−^ mice reverses weight gain and insulin resistance, while depletion of CD8^+^ T cells in adipose tissue reduces macrophage density and improves insulin sensitivity (41, 42).

Adoptive transfer models utilizing invariant natural killer T cells (iNKT) have demonstrated a role for these cells in modulating adipose tissue inflammation. NKT cells are lymphocytes that express NK cell surface markers and recognize CD1d (a non-polymorphic MHC I-like molecule) in its lipid-containing conformation (57). They are an important link between the innate and adaptive immune system. Type I NKT (iNKT) cells express a highly restricted (invariant) TCRα-chain (Vα24-Jα18 in humans paired with Vβ11 and Vα14-Jα18 paired with Vβ8.2, Vβ7, or Vβ2 in mice). Type II NKT cells on the other hand have diverse TCRs (58). Notably, the different TCRs expressed on type II NKT cells can recognize the same lipid antigens. In humans, type II NKT cells are more frequent than type I NKT cells, but the opposite is the case in mice (59, 60). Type II NKT cells have been shown to prevent high fat-induced obesity in some studies (61, 62), though they were shown to exacerbate diet-induced obesity in another (63). iNKT cells are highly plastic and can express both anti-inflammatory (61) and pro-inflammatory (64) cytokines depending on the activating stimuli. They recognize alpha-galactosylceramide and exert anti-inflammatory effects controlling the presence of Treg and macrophage populations in the adipose tissue in an IL-2- and IFN-γ-dependent manner (61, 65–68). Adipocytes can also serve as non-professional antigen presenting cells for iNKT cells with high levels of CD1d expression (69).

CD4^+^ iNKT cells express CXCR4 and CCR5 chemokine receptors and can be infected by HIV (70–72). Infection of CD4^+^ iNKT cells in humans and non-human primates leads to decline of CD4^+^ T cells. Couturier et al. compared NKT cells (CD3^+^/CD16^+^/CD27^+^/CD56^+^) in VAT of SHIV-SF162p3- infected macaques and healthy controls during chronic and acute infection, and found no differences in the percentage of these cells between groups (3). At present, there are no studies that assessed adipose tissue NKT cells in PLWH, and further studies are warranted to explore the role of NKT cells in tissue inflammation and metabolic disease (73).

### Macrophages in HIV/SIV and obesity

Progressive weight gain is accompanied by adipose tissue macrophage infiltration and inflammation (18, 41, 74–79). In obesity, hypertrophied adipocytes express increased macrophage inflammatory protein 1α (MIP-1α) and macrophage chemotactic protein 1 (MCP-1), which promote macrophage infiltration, and IL-8, which promotes neutrophil chemotaxis (80–82). Adipose tissue from obese individuals and animal models contains a greater density of macrophages with a pro-inflammatory M1 cytokine phenotype (characterized by high TNF-α, IL-6, IL-12, IL-23, and inducible nitric oxide synthase production). An environment high in these cytokines inhibits adipocyte insulin signaling by reducing expression of insulin receptor substrate-1 (IRS-1), phosphoinositide 3-kinase p85α, and glucose transporter type 4 (GLUT4) via cell surface receptors and other mechanisms (75, 83–86). The contribution of adipose tissue macrophages to metabolic disease is also supported by the higher levels of inflammatory cytokine and other protein expression in SAT from insulin resistant persons compared to insulin sensitive, including higher MCP-1, CD68, scavenger receptor A, visfatin, and oxidized LDL receptor 1 (87).

Studies of PLWH report conflicting results on adipose tissue macrophage changes compared to HIV-negative controls, but, in general, studies of PLWH have not demonstrated the marked enrichment in pro-inflammatory M1 macrophages observed in obesity. Peripheral lipoatrophy was accompanied by increased SAT macrophage density in one comparative study, in addition to increased fibrosis, apoptosis, and vessel density (88). In contrast, a study of gluteal fold adipose tissue (GFAT) found minimal differences in macrophage content (measured as cells/hpf) between PLWH with and without clinically-evident lipoatrophy, and HIV-negative controls, but higher IL-6, IL-8, IL-12p40, and MIP-1α, and lower interferon gamma and eotaxin levels, in adipose tissue supernatant from the PLWH (89). While the density of adipose tissue macrophages did not differ by lipoatrophy status in the PLWH, patients exposed to zidovudine and stavudine (older thymidine analog antiretroviral medications) had marginally higher density. Furthermore, median HIV DNA in circulating CD14^+^CD16^+^ monocytes, a subset more apt to be infected by HIV, was higher in the PLWH with clinically-evident lipoatrophy compared to those without lipoatrophy (89). HIV infection of CD14^+^CD16^+^ monocytes increases constitutive expression of pro-inflammatory cytokines, impairs phagocytic capacity, and increases antigen-stimulated cytokine expression (90–94). These findings suggest a role for adipose tissue macrophages in the development of lipoatrophy in PLWH, though the effect may stem from qualitative changes (e.g., greater cytokine expression) rather than greater density.

Adipose tissue CD3^−^/CD14^+^/HLA-DR^+^ macrophages represented a small proportion (<1–2%) of the total SVF cells in SIV-negative macaques, but were ~50% lower in acutely (4 weeks post-infection) SIV-infected macaques (3). In contrast, a study of macaques infected with SIV for a median of 15 months found a significantly higher proportion of macrophages among CD45+ cells in SAT, but not VAT, from SIV-infected animals compared to controls (2). Furthermore, the frequency of CD206^+^CD163^−^ macrophages (selected by the authors as markers of “M2” anti-inflammatory cells) was significantly lower in SIV-infected animals compared to non-infected animals in both SAT and VAT, while the proportion of CD206^−^CD163^−^ macrophages (thought to correspond to pro-inflammatory cells) was elevated in SIV-infected animals.

The relationship between CD8^+^ T cell enrichment and the accumulation of macrophages in adipose tissue in PLWH is an area for additional study, which will inform both our understanding of metabolic changes in HIV and the pathogenesis of adipose tissue inflammation more broadly. In the 2009 study by Nishimura et al. CD8^+^ T cell infiltration into adipose tissue in obese mice preceded the recruitment of M1-phenotype macrophages (41). The experimental depletion of CD8^+^ T cells in obese mice, and diet-induced obesity in *Cd8*^−^*/Cd8*^−^ knockout mice, was accompanied by reduced adipose tissue macrophage content and local inflammatory mediators, suggesting CD8^+^ T cells are both necessary and sufficient to promote macrophage recruitment in obesity. If HIV infection is characterized by adipose tissue CD8^+^ T cell enrichment without pronounced macrophage recruitment, this may reflect the need for a costimulatory factor in obese adipose tissue to recruit macrophages in the presence of CD8^+^ T cells, may indicate that cytokine expression profiles of adipose tissue CD8^+^ T cells differ in obesity and HIV infection, or reflect another as yet unknown mechanism.

### Studies in humanized mice

Several groups have used humanized mice to study HIV-1 tissue and cellular replication as well as immune responses during acute and chronic infections. Arainga et al. infected humanized mice with HIV-1 and examined tissue sites for viral infection. The authors assessed the blood, brain, gut, kidney, lungs, liver, lymph nodes, and spleen, and were able to detect HIV-1 integrated DNA and multi-spliced and unspliced RNA in different cell types, though adipose tissue was not evaluated (95). This study suggests that humanized mice infected with HIV and treated with ART might serve as a model to study the effects of chronic, treated HIV infection on metabolic status. A study by Cheng et al. evaluated humanized mice engrafted with hematopoietic stem cells (HSC), reconstituted with human lymphoid and myeloid lineages, and infected with HIV-1 before treatment with ART (96). The study showed a reduction of HIV replication to undetectable levels with treatment, but the persistence of HIV-1 in reservoirs and a viral rebound after stopping. At present, to our knowledge, no group has used humanized mice infected with HIV to explore adipose tissue biology.

## Adipose tissue: a viral reservoir

### Hiv and SIV

**Figure 2:**
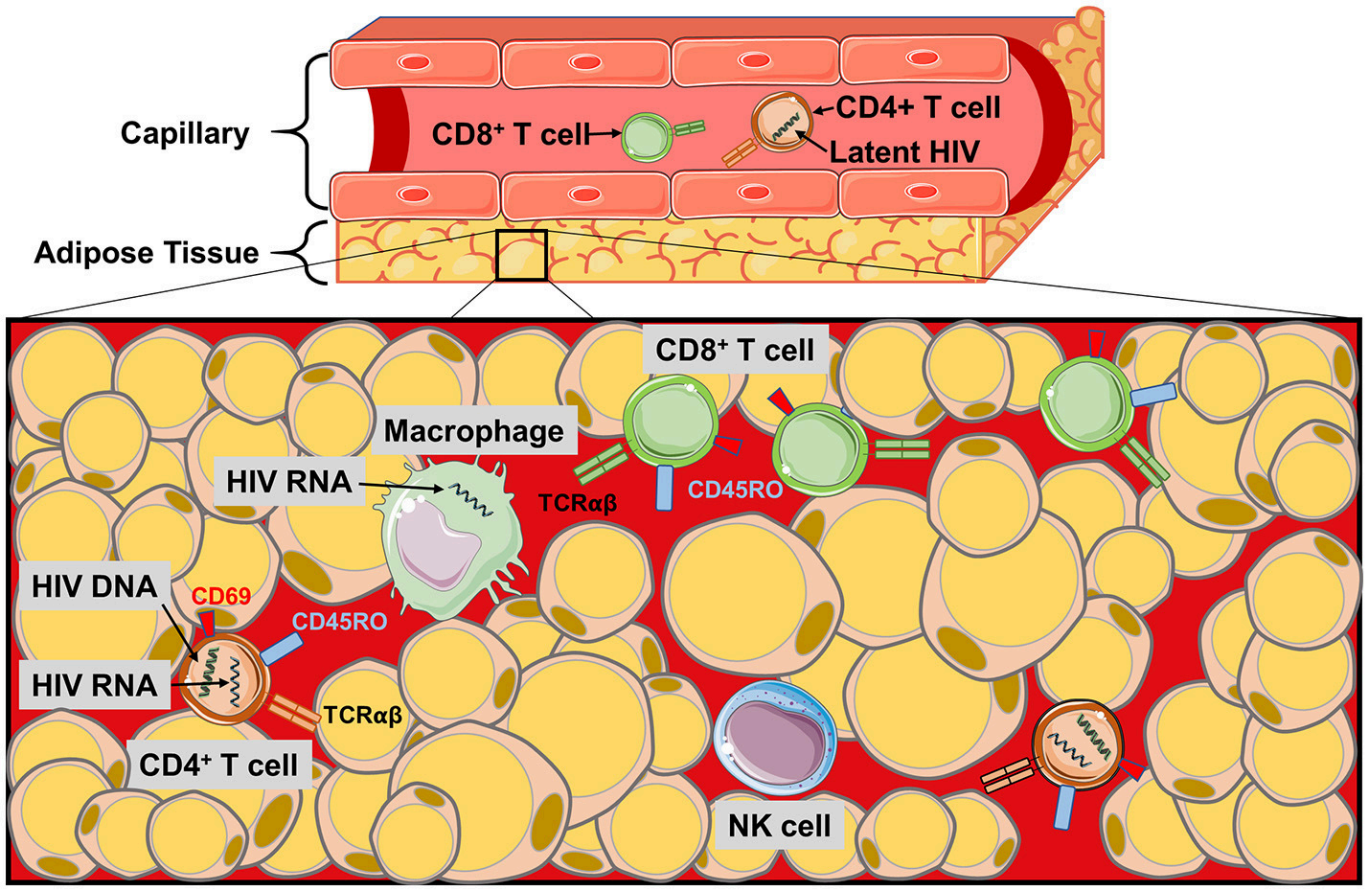
The HIV/SIV viral reservoir in adipose tissue. Schematic of the immune cell populations in PLWH and SIV-infected primates where latent and replicating HIV and SIV have been identified. HIV RNA and proviral DNA has been detected in the CD4^+^ T cells of the SVF, and also in macrophages during SIV infection. Poor perpetration of some antiretroviral agents into adipose tissue and the distance of CD4^+^ T cells from the pericapillary area may make targeting the latent reservoir of adipose CD4^+^ T cells difficult. Similarly, the localization of the CD8^+^ T cells to the pericapillary area may prevent cytotoxic killing of HIV-infected CD4^+^ T cells in the adipose tissue.

CD4^+^ T cells in adipose tissue are predominantly an activated memory phenotype (CD45RO^+^CD69^+^), which may serve as a reservoir for HIV persistence (1, 2, 19, 33, 97). Replication-competent HIV has been detected in sorted and *ex vivo* CD4^+^ T cells from the adipose tissue of aviremic, ART-treated patients, and in adipose-resident CD4^+^ T cells the median copy number of latent HIV proviral DNA (i.e., integrated into the host cell DNA) in adipose tissue CD4^+^ T cells was equivalent to circulating CD4^+^ T cells (2). Similarly, SVF cells from several adipose depots (visceral, subcutaneous, and deep neck) contained detectable HIV DNA at comparable frequencies to memory CD4^+^ T cells purified from peripheral blood, mesenteric lymph nodes, and thymus (1). Furthermore, SIV DNA and RNA were detected in SVF from SAT and VAT, sorted blood CD4^+^ T cells, and CD14^+^ macrophages in macaques (2, 3, 5). These findings indicate adipose tissue may serve as a HIV and SIV DNA reservoir, and a site for actively replicating (RNA-producing) virus. Viral persistence in adipose tissue may be partly due to inadequate distribution of ART. *In vitro* experiments using HIV-1 infected CD4^+^ T cells and primary human adipocytes revealed a reduced drug efficacy due to lower penetration of nucleoside/nucleotide reverse transcriptase inhibitors. Integrase inhibitors, on the other hand, penetrate adipose tissue (7). Figure 2 summarizes the major findings regarding HIV and SIV persistence in adipose tissue.

Adipose tissue latently-infected CD4^+^ T cells likely promote a local inflammatory environment. While the co-culture of preadipocytes or adipocytes with HIV-infected memory CD4^+^ T cells did not promote T cell activation or virus production, the addition of IL-2, IL-7, or IL-15, three cytokines present in adipose tissue (98–100), increased CD69 activation marker expression and p24 HIV antigen production by two-fold or more (1). Furthermore, preadipocyte IL-6 expression increased nearly three-fold in the presence of latently-infected CD4^+^ T cells (1). These findings may reflect a cycle in which an inflammatory adipose tissue environment promotes virus production and activation of latently-infected CD4^+^ T cells, and these CD4^+^ T cells in turn increase preadipocyte pro-inflammatory cytokine production (101–104).

Finally, if adipose tissue serves as a reservoir for latently-infected CD4^+^ T cells, these cells may be more protected from cytotoxic CD8^+^ T cells than those in circulation. This possibility is supported by studies in macaques showing CD4^+^ T cells are present deep in the adipose tissue while CD8^+^ T cells are generally localized to the pericapillary area (2). The development of therapies for eradication of the latent HIV reservoir will need to consider the potential challenges posed by adipose tissue CD4^+^ T cells.

### Non-retroviral reservoir

The presence of HIV and SIV proviral DNA and free RNA virus in adipose tissue is not unique to these two pathogens, and studies over the past 70 years have documented the infiltration of adipose tissue by a number of viruses spanning multiple Baltimore groupings. These pathogens are presumably engaged to varying degrees by the local immune system, resulting in changes in immune cell populations, expression of cytokines and other immune mediators, and effects on adipocyte energy storage and metabolic fitness.

Studies in the 1950s described the *in vivo* infection of adipose tissue and the adipotropism of Coxsackie virus (105), rabies (106), and polioviruses (107, 108). Later work in murine and primate models in the 1980s and 1990s described the infection of adipose tissue and subsequent metabolic and pathologic changes introduced by cytomegalovirus (109–111) and Ebola-Reston virus (112). More recent work has re-established adipose tissue as a key reservoir for influenza (113), the murid herpesviruses MHV68 and MHV4 (114, 115), and cytomegalovirus (116, 117). Epidemiologic studies have also linked untreated viral infections to changes in adipose tissue mass and metabolism, including childhood obesity associated with cytomegalovirus and total herpesvirus burden (118), and obesity and insulin resistance in chronic hepatitis C infection in adults (119).

## Conclusion

The multifaceted roles of adipose tissue as an energy storage depot, metabolic regulator, and endocrine organ occur against the backdrop of a complex mix of innate and adaptive immune cells interacting in an environment prone to infiltration by a range of viral pathogens. T cells are a major component of the adipose tissue milieu, and while the enrichment of CD8^+^ T cells appears broadly similar in obesity and HIV infection, differences in CD4^+^ T cells and, likely, macrophage populations suggest that the stimuli driving CD8^+^ cell enrichment and the interactions of CD8^+^ cells with other immune cells differs between the two conditions.

At this time, studies of T cell populations in HIV/SIV-infected subjects have only utilized surface marker phenotyping, and additional data on transcriptional profiles is needed to understand whether adipose tissue T cells in HIV/SIV and obesity are behaving in a similar manner, and whether adipose tissue T cells from PLWH have similar functional properties to T cells in the peripheral blood. Finally, HIV and SIV proviral DNA, and free RNA virus, can be detected in the SVF of VAT and SAT, and replication-competent HIV is found in CD4^+^ T cells. Evidence that adipose tissue is a viral reservoir for HIV and SIV suggests that some of the T cells within the adipose tissue may be virus-specific. Future studies to identify TCRs expressed by T cells within adipose tissue and their corresponding epitopes will help answer this question, and is currently being explored by our group. Finally, HIV-infected CD4^+^ T cells within adipose tissue may serve as a relatively protected viral compartment, which should be considered in future studies of interventions to deplete the HIV reservoir.

## Author contributions

All authors listed have made a substantial, direct and intellectual contribution to the work, and approved it for publication.

### Conflict of interest statement

The authors declare that the research was conducted in the absence of any commercial or financial relationships that could be construed as a potential conflict of interest.
